# Exploring the Association between Misinformation Endorsement, Opinions on the Government Response, Risk Perception, and COVID-19 Vaccine Hesitancy in the US, Canada, and Italy

**DOI:** 10.3390/vaccines10050671

**Published:** 2022-04-23

**Authors:** Elena Savoia, Nigel Walsh Harriman, Rachael Piltch-Loeb, Marco Bonetti, Veronica Toffolutti, Marcia A. Testa

**Affiliations:** 1 Emergency Preparedness Research, Evaluation & Practice Program, Harvard T.H. Chan School of Public Health, 677 Huntington Avenue, Boston, MA 02115, USA; esavoia@hsph.harvard.edu (E.S.); piltch-loeb@hsph.harvard.edu (R.P.-L.); testa@hsph.harvard.edu (M.A.T.); 2 Department of Biostatistics, Harvard T.H. Chan School of Public Health, 677 Huntington Avenue, Boston, MA 02115, USA; 3 Carlo F. Dondena Research Center and Covid Crisis Lab, Bocconi University, 20136 Milan, Italy; marco.bonetti@unibocconi.it; 4 Centre for Health Economics & Policy Innovation (CHEPI), Department of Economics & Public Policy, Imperial College, London SW7 2AZ, UK; v.toffolutti@imperial.ac.uk

**Keywords:** COVID-19 vaccine hesitancy, misinformation, government actions, communication

## Abstract

The COVID-19 pandemic has highlighted the adverse consequences created by an infodemic, specifically bringing attention to compliance with public health guidance and vaccine uptake. COVID-19 vaccine hesitancy is a complex construct that is related to health beliefs, misinformation exposure, and perceptions of governmental institutions. This study draws on theoretical models and current data on the COVID-19 infodemic to explore the association between the perceived risk of COVID-19, level of misinformation endorsement, and opinions about the government response on vaccine uptake. We surveyed a sample of 2697 respondents from the US, Canada, and Italy using a mobile platform between 21–28 May 2021. Using multivariate regression, we found that country of residence, risk perception of contracting and spreading COVID-19, perception of government response and transparency, and misinformation endorsement were associated with the odds of vaccine hesitancy. Higher perceived risk was associated with lower odds of hesitancy, while lower perceptions of government response and higher misinformation endorsement were associated with higher hesitancy.

## 1. Introduction

In the last twenty years, rapidly evolving technologies, including the use of mobile communications and social media platforms, have dramatically increased the complexity of the information ecosystem and altered the traditional means by which people access and share information. Such complexity has challenged governments’ capabilities in communicating to the public and managing infodemic-type situations, in which there is an overabundance of information—some accurate and some not—during an epidemic [1].

In this paper, we frame the phenomenon of infodemics, focusing on two specific aspects that occur during infodemics—misinformation endorsement and trust in government—as potential drivers of COVID-19 vaccine hesitancy. We ground our research in the Health Belief Model using an exploratory study design. 

Infodemics create a myriad of communication problems whereby individuals may not know what information is accurate or trustworthy and as such, mis- and disinformation can thrive. The COVID-19 pandemic has highlighted the negative consequences of an infodemic. Roozenbeek et al. demonstrated that increased susceptibility to misinformation reduces people’s compliance with public health guidance, as well as their intention to get vaccinated and recommend the vaccine to friends and family [2]. 

A study conducted in the United Kingdom and the United States quantified the impact of misinformation on vaccination intent and found that, in an experimental setting, exposure to misinformation decreased study participants’ self-reported intent to get the COVID-19 vaccine [3]. The phenomenon of vaccine hesitancy is widespread, and a varying amount of COVID-19 vaccine hesitancy is present in all countries [4]. Interestingly, the association between vaccine hesitancy and the actual risk of getting infected with SARS-CoV-2 is not clear [5,6]. A recent review of the literature on COVID-19 vaccine hesitancy showed a decline in vaccination intention in the USA and Italy during a time when the confirmed COVID-19 cases in both countries were among the ten highest in the world [7]. Another recent review discusses the possible detrimental global impact of myths and conspiracy theories related to COVID-19 and its vaccines on general and COVID-19-specific vaccine hesitancy [8].

Vaccinations have long been a central topic of the mis- and disinformation discourse, often as part of conspiracy theories where the sentiment of distrust in governments and healthcare institutions is part of the narration [9,10,11,12]. Such theories, once limited to fringe audiences, have now become commonplace in mass media, the internet, and social media [13]. Conspiracy theory creation thrives in both information voids and complex information ecosystems as audiences attempt to find explanations for missing, confusing, contradictory, or an overabundance of information they are experiencing [14,15]. This confusion is sometimes attributed to low scientific literacy but is also related to the contradictory messages released by government officials and scientists, which are likely to reduce the trust in those authorities [16]. In such a complex information environment, the public’s attention can easily shift from public health and/or government advice to alternative explanations that can negatively impact people’s actions. Consequently, vaccine hesitancy and lack of compliance with public health advice are not only fueled by the spread of misinformation, but also by distrust in scientific and government institutions [17,18,19,20,21]. Belief in misinformation or conspiratorial narratives may not be mutually exclusive from belief in the narrative reflecting scientific consensus [22]. 

Theories of health behavior identify a broad array of cognitive, social, and contextual factors that can influence vaccine hesitancy. The subjective risk perception of vaccine acceptance may be affected by psychological dispositions, fear of side effects and individualized lack of efficacy, health-related fears, beliefs and cognitions, and confirmatory biases involving pursuance of information congruous with one’s established beliefs about vaccines [23,24]. The Health Belief Model (HBM), which has been used to study COVID-19 vaccine hesitancy and attitudes towards vaccination requirements for travel, asserts that people weigh the severity of the health threat they confront (e.g., perceived risk of getting infected and sick from SARS-CoV2) and the perceived benefits or harms of taking a specific action (e.g., vaccination) [11,25,26,27,28]. It is also theorized that the individual assessment of one’s own risk can be influenced by the information received and the social context in which the individual lives and interacts [29]. It is well-established that many people seek information that supports their convictions—a phenomenon called “confirmation bias”—and that misinformation is frequently shared in echo chambers of like-minded groups of individuals [30,31,32]. As such, within these groups, the social expectation of not getting the vaccine may play a critical role in shaping vaccine intentions. 

COVID-19 vaccine hesitancy is a complex construct related to health beliefs, misinformation exposure, and perception of governmental institutions. This study draws on the existing theoretical literature and current information on the COVID-19 infodemic to explore the association between perceived risk of COVID-19, levels of misinformation endorsement, and opinions about the government response on vaccine uptake in a sample of over 2500 respondents from the US, Canada, and Italy.

## 2. Materials and Methods

### 2.1. Data Collection

We used a cross-sectional study design and collected data with an online survey. The survey was implemented via mobile phones using the survey platform Pollfish and was limited to individuals aged ≥ 18 residing in the US, Canada, and Italy [33]. A screening question was used to identify respondents who were not vaccinated or who had received only one dose of a COVID-19 vaccine requiring two doses. The Pollfish platform uses random device engagement (RDE), an approach similar to Random Device Dialing (RDD), to reach users engaged in using mobile applications (rather than calling them) who are identified only by a unique device ID. Compared to RDD, RDE results in a higher response rate and avoids the potential bias of interviewer–respondent interactions [34,35]. Like third-party advertising companies, Pollfish pays mobile application developers to display and promote the surveys to their users using crowdsourcing. Pollfish has over 1 billion registered users worldwide. For this survey, a random sample of users who fit the study’s eligibility criteria was initially selected and data were collected between 21–28 May 2021. The study protocol and survey instrument were approved by the Harvard T.H. Chan School of Public Health Institutional Review Board (IRB) on 8 December 2020 (protocol #20-203) and by the Bocconi University IRB on 22 April 2021 (protocol #31146). The respondents were asked to consent to participate in the study immediately before responding to the survey questions. The English version of the questionnaire is provided as Supplementary Material File S1 to this manuscript. Questions related to misinformation were created based on a previous analysis of frequently reported tropes and misinformation narratives [36]. We solicited feedback on the items from a small sample (*n* = 20) of individuals who spoke English, French, and/or Italian to determine if the items we developed measured our intended construct at face value and if the items could be intelligibly translated to French and/or Italian. The minor cognitive debriefing feedback we received regarding item re-wording was incorporated in a revised version of the questionnaire prior to its implementation. The survey was translated into Canadian French and Italian and back-translated into English for validation purposes. All samples had equally distributed quotas by sex and age groups and the Canadian sample was equally distributed between French and English speakers. In Canada, we sampled two different groups, one from the English-speaking part of the country and one from the French-speaking, to fully acknowledge the role that the two different cultures could play in vaccine hesitancy. Canadian respondents were given the option to respond in English or French and the datasets derived from the two samples were analyzed independently. As the minimum amount of time to thoughtfully complete the survey was tested to be three minutes, we used this time criteria as a method for data quality assurance and removed any questionnaire completed in less than three minutes.

### 2.2. Dependent Variable

The dependent variable of interest was COVID-19 vaccine hesitancy. It was measured by the response to the question: “*If you were offered a COVID-19 vaccine—at no cost to you—how likely are you to take it*?” Responses were as follows: (1) I would not take it at the moment but would consider it later on, (2) Very unlikely, (3) Somewhat unlikely, (4) I am not sure, (5) Somewhat likely, and (6) Very likely. For dichotomization of the dependent variable, the responses were coded as “not hesitant” if the answer “Very likely” was chosen, and as “hesitant” if any other category was chosen.

### 2.3. Independent Variables

Table 1 presents the list of independent variables and how responses were categorized. We collected data on age, sex, race, educational attainment, employment status, country of residence, risk perception of contracting and spreading COVID-19, opinions about the appropriateness of the government response in trying to curb the spread of the virus, experience in seeking government aid, and endorsement of COVID-19 vaccine beliefs related to misinformation. Participant race was not collected for the Italian sample. More specifically, participants were asked if they thought they were receiving transparent information about the COVID-19 situation from national government officials and their opinion about the appropriateness of the response measures taken by their country’s government up to the time of the survey (May 2021). Regarding their experience in seeking financial aid, respondents were asked to report if they had requested and received government aid since the start of the pandemic such as unemployment benefits, wage support, paid leave, business aid, and other types of aid (i.e., food, benefits, allowances). 

We measured COVID-19 risk perception using three questions that described situations related to the spread of the virus: contracting COVID-19 at work, contracting COVID-19 outside of work, and infecting your family or friends with COVID-19. For each question, respondents were asked to report their level of concern ranging from 1 (not concerned) to 3 (very concerned). We then performed a principal component factor analysis on the three items, found that there was one factor, and, as a result, summed the responses from each of the three questions to create a summative 3-item scale. This scale had values ranging from 3 to 9. From the sample’s responses obtained, we categorized the responses into quartiles and then created three levels of COVID-19 risk perception: low (<25th percentile), medium (≥25th percentile; <75th percentile), and high (≥75th percentile), with the “medium” category intentionally twice as large as the “low” and “high” categories to capture the more extreme responses.

To operationalize the construct of COVID-19 vaccine misinformation endorsement, respondents were asked to indicate how much they agreed with seven statements on a 7-point Likert scale ranging from 1 (strongly disagree) to 7 (strongly agree). The seven statements referred to misinformation such as the belief that: you can get COVID-19 from the vaccine, you can get other diseases from the vaccine, the vaccine contains toxic ingredients that can harm your health, the vaccine can mess up your DNA, the vaccine can cause infertility, the fast production of the vaccine compromised its safety, and that it contains a microchip with tracking capabilities. The questions were negatively worded to avoid spreading misinformation using the survey itself and subsequently reverse-coded so that a higher score indicated greater misinformation endorsement. Following principal component factor analysis to confirm unidimensionality, we created a summative scale by adding the responses to the seven statements. The scale had values ranging from 7 to 49, with higher scores indicating stronger endorsement. The summative score was then categorized into quartiles to create four levels of misinformation endorsement: low (<25th percentile), medium–low (≥25th percentile; <50th percentile), medium–high (≥50th percentile; <75th percentile), and high (≥75th percentile).

### 2.4. Statistical Analyses

We first computed descriptive statistics and performed Chi-squared tests to determine if the distributions of the dependent and independent variables differed by country. We then performed a principal component factor analysis to explore the factor structure of the risk perception and misinformation endorsement scales. Following this, we utilized nested hierarchical logistic regression models to investigate the predictors of COVID-19 vaccine hesitancy with the following methodology: model 1 estimated the association between hesitancy and socio-demographic variables (age, sex, education attainment, employment status, and country of residence—with the additional differentiation of preferred language for Canada) and COVID-19 risk perception. Model 2 included all the parameters from model 1 and added three variables related to the perception of government response and request for aid. Model 3 then added a nominal variable for the quartiles in the misinformation endorsement scale. Finally, we tested for the interaction between the perception of government transparency and misinformation endorsement.

To explore the differences and similarities in the results by country, we repeated the analyses presented in model 3 separately for each of the three countries: United States (*n* = 726), Canada (*n* = 985), and Italy (*n* = 984). We included a variable for the respondent’s race in the analyses that were restricted to the United States and Canada, which had similar race categories based on census definitions.

Model fit was assessed by performing the Hosmer–Lemeshow Goodness-of-Fit test and model discrimination was assessed with the area under the ROC curve [37,38,39]. *P*-values corresponding to multivariate Wald test statistics were used to assess the significance of the independent variables, and a likelihood ratio test was used to assess the significance of the interaction between the perception of government transparency and misinformation endorsement. The alpha value for significance was set at 0.05. Data were analyzed using STATA (version 15).

## 3. Results

### 3.1. Sample Characteristics

Descriptive statistics are presented in Table 1. We collected data from 2750 respondents, of which 1000 respondents were from Canada (50% French-speaking by sample design), 1000 from Italy, and 750 from the USA. Fifty-three questionnaires (2% of the sample) were removed from the sample due to a completion time of less than 3 min, leaving 2697 completed questionnaires suitable for data analysis. The sample was balanced by design in terms of age and sex, as described in the methods. Except for age, sex, and employment status, the distribution of all variables varied by country (Chi-square, *p* < 0.001). Participant race was only assessed in the United States and Canada—the majority of respondents from these two countries (67%) identified as non-Hispanic white. The plurality of the sample had attained a high school or equivalent level of education (31%), 14% had attained a post-bachelor’s degree, and 7% had less than a high school degree. The US sample had the greatest proportion of respondents holding a post-bachelor’s degree (30%), while Italy had the greatest proportion of respondents whose level of education was less than high school (8%). Employment status did not vary by country, and, overall, 62% of respondents were employed. Overall, 35% of respondents said they had not received the vaccine yet and were not scheduled for an appointment. When asked if they would take the vaccine if offered to them at no cost, 48% of respondents expressed some hesitation. The sample of respondents living in the US had the greatest proportion of vaccine hesitancy (63%), followed by the Italian respondents (43%) and then the Canadians (42%).

### 3.2. COVID-19 Risk Perception

A Bartlett’s test of sphericity (*p* < 0.001) and Kaiser–Meyer–Olkin (KMO = 0.7) measure of sampling adequacy indicated the three items related to COVID-19 risk perception were suitable for factor analysis. When we repeated the measures among the country samples—Canada and Italy both had a KMO of 0.69 and the US KMO was 0.71. In the overall sample, a principal component factor analysis of the three items resulted in one factor with an eigenvalue greater than one, thereby explaining 69.9% of the variance. All factor loadings of the three questions were over 0.8. We obtained similar results when repeating the factor analysis within each country subsample—all three questions loaded onto one factor with an eigenvalue greater than one, and all factor loadings were equal to or above 0.8. Cronbach alpha values for the items were as follows: entire sample = 0.78; US = 0.8; Canada = 0.77; and Italy = 0.78. After summing the responses for the three items, the resulting score had a mean of 6.4 (SD 1.9) and a median of 7 (range 3–9). Our final COVID-19 risk perception variable was developed by categorizing the sample responses based on quartiles and then re-categorizing the quartiles into three levels as follows: low (<25th percentile), medium (≥25th percentile; <75th percentile), and high (≥75th percentile), as mentioned previously in the Section 2. The Chi-squared results indicated that the distribution of risk perception varied significantly by country (*p* < 0.001). The United States had the highest percentage of respondents with low COVID-19 risk perception (25%), while Italy had the lowest (10%). Both the US and Italy had higher percentages of respondents with high COVID-19 risk perception (35% each) than Canada (27%).

### 3.3. Perception of Government Response and Request for Government Aid

Overall, 56% of respondents felt the measures undertaken by their government to control the pandemic were not effective. American respondents had the highest proportion of individuals believing the measures were ineffective (67%). In terms of requests for government aid, 18% of respondents reported they applied for government aid during the pandemic and at least one or more requests of theirs were rejected. Italy and Canada had similar percentages of respondents whose requests for aid were rejected (11% and 13%, respectively), while the US had the highest percentage (34%). Most respondents believed their government had been sharing transparent information with the public about the COVID-19 situation throughout the course of the pandemic (68%), however, American respondents were less likely to believe that their government had been transparent (39%).

### 3.4. Misinformation Endorsement

A Bartlett’s test of sphericity (*p* < 0.001) and Kaiser–Meyer–Olkin (KMO > 0.8) measure of sampling adequacy indicated that the seven statements used to describe misinformation endorsement were suitable for factor analysis among the entire sample, as well as for each country-specific subsample. A principal component factor analysis of the seven items resulted in one factor with an eigenvalue greater than one, thereby explaining 51.4% of the variance. All factor loadings of the seven questions were over 0.6. We obtained similar results when repeating the factor analysis within each country subsample—all seven questions loaded onto one factor with an eigenvalue greater than one, and all factor loadings were above 0.5. Cronbach alpha values for the items were as follows: entire sample = 0.84; US = 0.88; Canada = 0.87; and Italy = 0.73. The resultant summative score had a mean of 24.2 (SD 9.1) and a median of 25 (range 7–49), with higher scores indicating stronger misinformation endorsement. Detailed descriptions of the factor analysis results can be found in the Supplementary Material Table S1. Two percent of the sample indicated that they strongly agreed with all statements, which would indicate no misinformation endorsement. After the score was divided into quartiles, Chi-squared tests indicated significant differences by country (*p* < 0.001). The US had the highest proportion of respondents in the fourth quartile (35% in the strongest endorsement group), while Italy had the lowest at 20%. Canada had the highest proportion of respondents in the first quartile (29% in the weakest endorsement group), while the US had the lowest at 21%.

### 3.5. Multivariable Analysis

In Table 2, we present the results of the nested hierarchical logistic regression models for COVID-19 vaccine hesitancy in our overall sample.

In Model 1, Wald tests indicated that the odds of vaccine hesitancy varied by age, sex, educational attainment, country, and risk perception of contracting and spreading COVID-19. Except for sex, these variables remained associated with vaccine hesitancy across all models. Specifically, we observed that participants who were 55 and older had 52% reduced odds of being vaccine-hesitant compared to those aged 18–24 (OR = 0.48, 95% CI: 0.37–0.63). Across all models, this association was confirmed, with little change in its magnitude and direction. Concerning participants’ sex, the results indicated that females had 22% increased odds of vaccine hesitancy compared to males (OR = 1.22, 95% CI: 1.03–1.44). Compared to participants with lower than high school education, those with a bachelor’s degree (OR = 0.67, 95% CI: 0.47–0.95) and post-bachelor’s degree (OR = 0.34, 95% CI: 0.23–0.5) had lower odds of vaccine hesitancy. When taking into consideration the language preferred by the respondent to complete the questionnaire, the results indicated that English-speaking respondents in Canada (OR = 0.35, 95% CI: 0.27–0.45), French-speaking respondents in Canada (OR = 0.31, 95% CI: 0.24–0.4), and Italian respondents (OR = 0.36, 95% CI: 0.29–0.45) had lower odds of vaccine hesitancy than US residents. This association persisted across all subsequent models, with little change to its magnitude. Finally, we observed a graded negative association between COVID-19 risk perception and the odds of vaccine hesitancy—compared to those with low COVID-19 risk perception, those with medium risk perception (OR = 0.44, 95% CI: 0.35–0.56), and high-risk perception (OR = 0.25, 95% CI: 0.19–0.32) had lower odds of being hesitant. Again, we note that there was little change to these estimates in all subsequent models.

In Model 2, we added three more variables to Model 1: perception of government measures to respond to the pandemic, perception of government transparency, and request for government aid. In this model, we no longer observed a significant difference in the odds of vaccine hesitancy by sex. Additionally, the difference in odds of being hesitant between those with a bachelor’s degree and those with less than high school education was no longer significant.

We continued to observe differences by age, country of residence and language, education status (post-bachelor’s degree vs. less than high school), and risk perception of contracting and spreading COVID-19 with little change to the associations described above. All three of the variables added to Model 2 were associated with vaccine hesitancy. The respondents who believed that the government response was either excessive, not useful, counter-productive, or were unsure about its effectiveness had 3.04 times the odds of being hesitant compared to those who believed the measures were “just right” (OR = 3.04, 95% CI: 2.54–3.64). Those who had at least one request for aid rejected by their government had 51% increased odds of being hesitant compared to those who had not had a request submitted or rejected (OR = 1.51, 95% CI: 1.19–1.93). Finally, participants who believed the government had not been sharing transparent information about the COVID-19 situation with the public had 88% increased odds of being hesitant compared to those who believed the government had shared transparent information (OR = 1.88, 95% CI: 1.55–2.28).

Model 3 included all the parameters from Model 2 with the addition of a nominal variable for the misinformation endorsement scale quartile. The Hosmer–Lemeshow Goodness-of-Fit test confirmed that the model was a good fit for the data (*p* = 0.248), and the area under the ROC curve for this model was 0.832 [37,38,39]. With the addition of misinformation endorsement, we observed that the difference in the odds of vaccine hesitancy among those with a post-bachelor’s degree and those with less than high school education was no longer significant. Additionally, having a request for aid rejected by the government was no longer associated with vaccine hesitancy.

In Model 3, age, country of residence and language, risk perception of contracting and spreading COVID-19, perception of government response and transparency, and misinformation endorsement were associated with the odds of vaccine hesitancy. Specifically, respondents aged over 55 had 39% decreased odds of being hesitant compared to respondents aged 18–24 (OR = 0.61, 95% CI 0.45–0.82). English-speaking respondents from Canada (OR = 0.41, 95% CI: 0.31–0.55), French-speaking respondents from Canada (OR = 0.48, 95% CI: 0.35–0.65), and Italian respondents (OR = 0.52, 95% CI: 0.4–0.67) all had lower odds of vaccine hesitancy than respondents from the United States. The respondents in the medium category of risk perception had 34% decreased odds of being hesitant compared to respondents in the lowest category of risk perception (OR = 0.66, 95% CI 0.5–0.87), and those in the highest category of risk perception had 63% decreased odds of being hesitant compared to those in the lowest category of risk perception (OR = 0.37, 95% CI 0.27–0.5). The respondents who believed that the government response was either excessive, not useful, counter-productive, or were unsure about its effectiveness had 144% increased odds of being hesitant about the vaccine compared to those who thought the government response was “just right” (OR = 2.44, 95% CI 2.01–2.96). Similarly, those who believed the government had not shared transparent information about the COVID-19 situation with the public had 35% increased odds (OR = 1.35, 95% CI 1.09–1.67) of being vaccine-hesitant, compared to those who believed the government had shared transparent information.

In Model 3, we found a dose-response relationship between misinformation endorsement and vaccine hesitancy. The respondents in the second quartile of the level of misinformation endorsement had 3.76 times the odds of being vaccine-hesitant compared to those in the first quartile—the lowest level of endorsement (OR = 3.76, 95% CI 2.79–5.05). Those in the third quartile had 9.82 times the odds of being vaccine-hesitant compared to those in the first quartile (OR = 9.82, 95% CI 7.3–13.23), and, finally, those in the fourth quartile (highest level of endorsement) had 13.68 times the odds of being vaccine-hesitant compared to those in the first quartile (OR = 13.68, 95% CI 10.01–18.7). No interaction was found between the perception of government transparency and misinformation endorsement.

### 3.6. Multivariable Analysis by Country

In Table 3, we present the results of the final model (Model 3) in each of the three countries we collected responses from: the United States (*n* = 726), Canada (*n* = 985), and Italy (*n* = 984). We included a variable for the respondent’s race in the analyses that were restricted to the United States and Canada. Within all three countries, the following variables were associated with vaccine hesitancy: risk perception of contracting and spreading COVID-19, perception of government measures to respond to the pandemic, and misinformation endorsement. Please refer to Table 3 for country-specific point estimates. Specifically, in each country, the respondents in the highest category of risk perception had lower odds of being hesitant compared to those in the lowest category of risk perception. The respondents who believed that the government response was either excessive, not useful, counter-productive, or were unsure about its effectiveness had higher odds of being vaccine-hesitant compared to those who thought the government response was “just right”. Within each country, we observed a dose-response relationship between misinformation endorsement and the odds of vaccine hesitancy. In contrast to the aggregated results, within each country, both educational attainment and the perception that the government was sharing transparent information were not associated with vaccine hesitancy.

We also noted the following differences between countries. In the United States, respondents who were aged 35–44 had 70% decreased odds of being vaccine-hesitant compared to those aged 18–24 (OR = 0.3, 95% CI 0.15–0.61), while respondents in Canada who were 55 and older had 69% decreased odds of being vaccine-hesitant compared to those who were 18–24 (OR = 0.31, 95% CI 0.17–0.56). In Canada, compared to non-Hispanic white respondents, Asian respondents had 52% decreased odds of being vaccine-hesitant (OR = 0.48, 95% CI 0.29–0.81). Finally, Italy was the only country in which having a request for aid rejected by the government was associated with vaccine hesitancy. Those who had at least one request for aid rejected had 73% increased odds of being vaccine-hesitant compared to those who had not had a request submitted or rejected (OR = 1.73, 95% CI: 1.1–2.73).

## 4. Discussion

Our study sought to explore and describe the determinants of vaccine hesitancy among a sample of unvaccinated and partially vaccinated individuals in the United States, Canada, and Italy. In our final logistic regression models, we found that increased age and risk perception were associated with lower odds of being vaccine-hesitant. Conversely, individuals who did not believe their government had responded appropriately to the pandemic or that the government was not sharing transparent information had higher odds of being vaccine-hesitant. Furthermore, individuals who endorsed COVID-19 vaccine misinformation had higher odds of being vaccine-hesitant. Finally, compared to those from Canada and Italy, respondents from the United States had higher odds of being vaccine-hesitant.

Our results are consistent with previous research demonstrating that risk perception about the harm caused by COVID-19 is a strong predictor of vaccine acceptance [40,41,42]. Risk perception, or an individual’s perceived susceptibility to a threat, is a key component of many health behavior change theories, and, overall, communication strategies that successfully influence risk perception can result in populations being more compliant with recommended behaviors. Based on our survey results, individuals with a high risk perception of getting sick from COVID-19 were less likely to be vaccine-hesitant and this result was consistent within each of the three countries examined in this study.

However, risk perception is only one of the many factors related to vaccine hesitancy. The construct of trust, for example, has also been studied as another important predictor of compliance with recommended COVID-19 public health measures, including the vaccine. Our results show that individuals who believed that measures adopted by the government had not been “right” and that the government had not been transparent in communicating with the public were more likely to be vaccine-hesitant compared to those with a favorable opinion about their government’s response. Building a trusted relationship with the public is critical to implementing successful public health interventions. Existing research has indicated that countries with higher levels of social and government trust have typically seen lower mortality rates [43]. A recent analysis of communication efforts across the Group of Seven (G7) countries (Canada, France, Germany, Italy, Japan, the United Kingdom, and the United States) revealed that trusted sources of information strongly predicted public attitudes toward protective measures promoted by the government [44]. In parallel, recent evidence from the United States and the United Kingdom shows that political trust appeared to be highly volatile and fragmented at the local level during the COVID-19 pandemic and that it depended on the level of reliance on scientific advice [45,46]. In the United States, the level of governmental distrust was already high before the pandemic, and the low reliance on science at the federal level might explain why governmental distrust is so high in this sample [45]. In our study, distrust in the government response in Italy is coupled with higher levels of vaccine hesitancy among those individuals who requested financial aid during the pandemic, but their application was rejected. This is in line with previous literature that shows that political trust is strongly related to welfare measures. Specifically, political dissatisfaction increased between 2008 and 2016, which was subsequent to welfare retrenchment measures of several governments in Europe [47]. In addition, Italy was one of the first countries to implement a vaccine mandate with the “green pass policy”. In that regard, recent research suggests the need for more public education and better communication when compulsory measures are being implemented [48].

In the COVID-19 vaccine hesitancy literature, trust has been analyzed across different dimensions, such as trust in the information being disseminated, trust in the messengers (i.e., health officials), trust in healthcare providers, trust in vaccine developers, and trust in research in general [6,49,50]. Our study contributes to the international literature by examining the construct from a different perspective compared to previous studies, focusing on opinions about the adequacy of the measures the government adopted to respond to COVID-19 and the transparency of government communication efforts.

The third area of our analysis was related to the effect of the infodemic, and specifically misinformation. Our data show an interesting “infodemic” phenomenon with a dose-response relationship between levels of misinformation endorsement and vaccine hesitancy. These results support the idea that not only the type of misinformation an individual believes may harm compliance with recommended behaviors, but also the amount of misinformation the individual endorses. The COVID-19 pandemic has highlighted the importance of infodemic management, government-level functions necessary to maintain communication effectiveness in promoting healthy behaviors, and mitigating the harm caused by mis- and disinformation [9,51]. However, data from a recent Organization for Economic Co-operation and Development (OECD) report on public communication states that only 38% of Centre of Governments have developed guidance related to the management of mis- and disinformation, indicating that governments may be inadequately prepared to address this phenomenon and that future plans need to focus on this important capability [52]. The exponential increase in the availability of mobile communications in the past two decades is another key driver in the rise of infodemics. While this study attempted to elucidate the role that COVID-19 misinformation endorsement (independent of source) plays in determining vaccine hesitancy, future research should address how the effects of misinformation endorsement may vary with information channel sources, such as social media, news media, or friends and family.

We recognize several limitations in our study. First, we do not know the extent to which expressed intent to take a vaccine is associated with actual behavior and, as such, we are unable to validate our results in terms of the impact of the variables we found to be associated with intention to get vaccinated compared to actual behaviors. Second, the cross-sectional study design is a key limitation for the conclusions concerning the explanatory variables, as such, we do not know whether the beliefs, attitudes, and opinions we measured are causally related to willingness to take the vaccine. Third, measuring misinformation is not a standardized process. We used specific beliefs and statements to conceptualize this construct, but misinformation evolves as science develops and new beliefs and uncertainties are expressed in the online and offline space. Finally, because our sample was not representative of the US, Canada, or Italy, it is important to acknowledge that these results are not necessarily generalizable outside of the sample, and that selection bias may have also influenced the results. Despite the study’s limitations, we believe our results are informative in supporting the need for enhancing governments’ capabilities in addressing mis- and disinformation. As part of such capabilities, our results suggest that being transparent in the communication process, acknowledging what is unknown about the safety and effectiveness of an intervention, detailing how decisions are made, and managing expectations about the government response may have a positive impact in enhancing the population’s compliance with recommended behaviors.

## 5. Conclusions

We found a dose-response relationship between misinformation endorsement and vaccine hesitancy, with individuals endorsing a greater amount of incorrect information being more likely to be vaccine-hesitant. Individuals of younger ages, those with lower risk perception of contracting and spreading COVID-19, those with negative opinions about the effectiveness of the government response, and about government transparency in communication efforts were also more likely to be vaccine-hesitant. Our results suggest that being transparent in the communication process and managing expectations about the government response may have a positive impact on the population’s compliance with COVID-19 vaccine uptake.

## Figures and Tables

**Table 1:** Descriptive Statistics (Overall and by Country).

Variable	United States (*n* = 726)		Canada (*n* = 985)		Italy (*n* = 986)		Total (*n* = 2697)		Country χ^2^ *p*-Value
**COVID-19 Vaccine Hesitancy**									
Non-Hesitant	268	37%	572	58%	560	57%	1400	52%	<0.001
Hesitant	458	63%	413	42%	426	43%	1297	48%	
**Age Category (balanced by design)**									
18–24	141	19%	198	20%	194	20%	533	20%	N/A ^b^
25–34	147	20%	193	20%	196	20%	536	20%	
35–44	143	20%	196	20%	198	20%	537	20%	
45–54	149	21%	199	20%	198	20%	546	20%	
Over 54	146	20%	199	20%	200	20%	545	20%	
**Sex (balanced by design)**									
Male	364	50%	491	50%	489	50%	1344	50%	N/A ^b^
Female	362	50%	494	50%	497	50%	1353	50%	
**Race ^a^**									
White, Non-Hispanic	463	64%	677	69%	-	-	1140	67%	<0.001
Black, Non-Hispanic	63	9%	52	5%	-	-	115	7%	
Asian	45	6%	128	13%	-	-	173	10%	
Hispanic	62	9%	14	1%	-	-	76	4%	
Two or more/Other/Prefer not to say	93	13%	114	12%	-	-	207	12%	
**Education Category**									
Less than high school	49	7%	49	5%	83	8%	181	7%	<0.001
High school or equivalent	162	22%	238	24%	440	45%	840	31%	
Some college	140	19%	276	28%	133	13%	549	20%	
Bachelor’s degree	132	18%	296	30%	277	28%	705	26%	
Post-graduate degree	221	30%	118	12%	51	5%	390	14%	
Other	22	3%	8	1%	2	0%	32	1%	
**Employment status**									
Not employed (includes students and retired individuals)	290	40%	370	38%	353	36%	1013	38%	0.216
Employed	436	60%	615	62%	633	64%	1684	62%	
**COVID-19 Risk Perception**									
Low COVID-19 Risk Perception (<25th percentile)	178	25%	215	22%	94	10%	487	18%	<0.001
Medium COVID-19 Risk Perception (≥25th percentile; <75th percentile)	294	40%	508	52%	548	56%	1350	50%	
High COVID-19 Risk Perception (≤75th percentile)	254	35%	262	27%	344	35%	860	32%	
**Perception of government response measures**									
Government measures just right	239	33%	469	48%	469	48%	1177	44%	<0.001
Government measures not right	487	67%	516	52%	517	52%	1520	56%	
**Request for government aid**									
No requests for government aid rejected or requested	482	66%	858	87%	877	89%	2217	82%	<0.001
At least one request rejected	244	34%	127	13%	109	11%	480	18%	
**Perception of government transparency**									
Government has been transparent	441	61%	653	66%	743	75%	1837	68%	<0.001
Government has not been transparent	285	39%	332	34%	243	25%	860	32%	
**Misinformation Endorsement Scale Quartile**									
Low Misinformation Endorsement (<25th percentile)	155	21%	283	29%	226	23%	664	25%	<0.001
Medium-Low (≥25th percentile; <50th percentile)	116	16%	233	24%	308	31%	657	24%	
Medium-High (≥50th percentile; <75th percentile)	198	27%	242	25%	251	25%	691	26%	
High Misinformation Endorsement (≥75th percentile)	257	35%	227	23%	201	20%	685	25%	

^a^ Race was not assessed in the Italian Survey. ^b^ N/A—χ^2^ Analysis “Not applicable” because survey was balanced by design.

**Table 2:** Nested Logistic Regressions for Vaccine Hesitancy in the Overall Sample (*n* = 2697).

	Model 1—Socio-Demographics & Risk Perception				Model 2—Model 1 + Government Perceptions				Model 3—Model 2 + Misinformation Endorsement			
	N		2697		N		2697		N		2697	
	Pseudo R^2^		0.0929		Pseudo R^2^		0.162		Pseudo R^2^		0.268	
VARIABLES	OR	SE	95% CI	Wald Test *p*-value	OR	SE	95% CI	Wald Test *p*-value	OR	SE	95% CI	Wald Test *p*-value
**Age group**			<0.001					0.004				0.018
18–24	*ref*				*ref*				*ref*			
25–34	0.88	0.12	(0.68–1.13)		0.94	0.13	(0.71–1.23)		0.9	0.14	(0.67–1.21)	
35–44	0.84	0.11	(0.64–1.09)		0.92	0.13	(0.7–1.21)		0.8	0.12	(0.6–1.08)	
45–54	0.79	0.11	(0.61–1.03)		0.97	0.14	(0.74–1.28)		0.88	0.13	(0.65–1.18)	
55+	0.48 **	0.06	(0.37–0.63)		0.62 **	0.09	(0.47–0.81)		0.61 **	0.09	(0.45–0.82)	
**Sex**			0.020					0.122				0.544
Male	*ref*				*ref*				*ref*			
Female	1.22 *	0.1	(1.03–1.44)		1.15	0.1	(0.96–1.37)		1.06	0.1	(0.88–1.28)	
**Educational attainment**			<0.001					<0.001				0.027
Less than high school	*ref*				*ref*				*ref*			
High school or equivalent	0.96	0.17	(0.68–1.35)		1.04	0.19	(0.72–1.49)		1.22	0.24	(0.83–1.78)	
Some college	0.7	0.13	(0.49–1)		0.82	0.16	(0.56–1.2)		1.13	0.24	(0.76–1.7)	
Bachelor’s degree	0.67 *	0.12	(0.47–0.95)		0.79	0.15	(0.54–1.14)		1.15	0.23	(0.78–1.71)	
Post-Bachelor’s degree	0.34 **	0.07	(0.23–0.5)		0.40 **	0.09	(0.26–0.61)		0.73	0.17	(0.47–1.14)	
Other	2.41	1.28	(0.85–6.82)		2.14	1.16	(0.74–6.22)		2.42	1.37	(0.8–7.33)	
**Employment status**			0.410					0.632				0.744
Not employed (includes students and retired)	*ref*				*ref*				*ref*			
Employed	0.93	0.09	(0.77–1.11)		0.95	0.09	(0.79–1.16)		0.97	0.1	(0.79–1.19)	
**Country of residence and language**			<0.001					<0.001				<0.001
Residents in US	*ref*				*ref*				*ref*			
Residents in Canada-English speaking	0.35 **	0.05	(0.27–0.45)		0.38 **	0.05	(0.29–0.49)		0.41 **	0.06	(0.31–0.55)	
Residents in Canada-French speaking	0.31 **	0.04	(0.24–0.4)		0.43 **	0.06	(0.33–0.57)		0.48 **	0.07	(0.35–0.65)	
Residents in Italy	0.36 **	0.04	(0.29–0.45)		0.47 **	0.06	(0.37–0.6)		0.52 **	0.07	(0.4–0.67)	
**COVID-19 risk perception**			<0.001					<0.001				<0.001
Low COVID-19 risk perception (<25th percentile)	*ref*				*ref*				*ref*			
Medium COVID-19 risk perception (≥25th percentile; <75th percentile)	0.44 **	0.05	(0.35–0.56)		0.59 **	0.08	(0.46–0.76)		0.66 **	0.09	(0.5–0.87)	
High COVID-19 risk perception (≤75th percentile)	0.25 **	0.03	(0.19–0.32)		0.35 **	0.05	(0.26–0.46)		0.37 **	0.06	(0.27–0.5)	
**Perception of government measures to respond to the pandemic**								<0.001				<0.001
Government measures just right					*ref*				*ref*			
Government measures have not been right					3.04 **	0.28	(2.54–3.64)		2.44 **	0.24	(2.01–2.96)	
**Request for government aid**								0.001				0.076
No requests rejected or applied for					*ref*				*ref*			
At least one request was rejected					1.51 **	0.19	(1.19–1.93)		1.27	0.17	(0.98–1.64)	
**Perception of government transparency**								<0.001				0.005
Government has been transparent					*ref*				*ref*			
Government has not been transparent					1.88 **	0.19	(1.55–2.28)		1.35 **	0.15	(1.09–1.67)	
**Misinformation endorsement**												<0.001
Low misinformation endorsement (<25th percentile)									*ref*			
Medium-Low (≥25th percentile; <50th percentile)									3.76 **	0.57	(2.79–5.05)	
Medium-High (≥50th percentile; <75th percentile)									9.82 **	1.49	(7.3–13.23)	
High misinformation endorsement (≥75th percentile)									13.68 **	2.18	(10.01–18.7)	

* The marginal test associated with the coefficient produced a *p*-value between 0.01 and 0.05. ** The marginal test associated with the coefficient produced a *p*-value smaller than 0.01.

**Table 3:** Final Model (Model 3) in United States, Canada, and Italy.

	United States				Canada				Italy			
	N		726		N		985		N		984	
	Pseudo R^2^		0.398		Pseudo R^2^		0.326		Pseudo R^2^		0.167	
VARIABLES	OR	SE	95% CI	Wald Test *p*-value	OR	SE	95% CI	Wald Test *p*-value	OR	SE	95% CI	Wald Test *p*-value
**Age group**				0.008				<0.001				0.182
18–24	*ref*				*ref*				*ref*			
25–34	0.66	0.23	(0.33–1.32)		1.08	0.28	(0.65–1.78)		0.97	0.23	(0.6–1.55)	
35–44	0.30 **	0.11	(0.15–0.61)		1.11	0.29	(0.67–1.85)		1.21	0.29	(0.76–1.94)	
45–54	0.76	0.27	(0.38–1.51)		0.74	0.2	(0.43–1.26)		1.31	0.32	(0.81–2.12)	
55+	0.96	0.36	(0.46–2)		0.31 **	0.09	(0.17–0.56)		0.77	0.19	(0.48–1.25)	
**Sex**				0.253				0.687				0.313
Male	*ref*				*ref*				*ref*			
Female	1.3	0.3	(0.83–2.05)		0.93	0.16	(0.67–1.3)		0.85	0.13	(0.63–1.16)	
**Race**				0.198				0.016				
White, Non-Hispanic	*ref*				*ref*							
Black, Non-Hispanic	2.06	0.79	(0.97–4.37)		1.31	0.47	(0.65–2.65)					
Asian	1.81	0.85	(0.73–4.53)		0.48 **	0.13	(0.29–0.81)			^a^		
Hispanic	1.88	0.72	(0.88–4)		0.97	0.64	(0.26–3.56)					
Two or more/Other/Prefer not to say	1.62	0.57	(0.81–3.23)		1.36	0.37	(0.8–2.32)					
**Educational attainment**				0.100				0.954				0.866
Less than high school	*ref*				*ref*				*ref*			
High school or equivalent	2.81 *	1.25	(1.18–6.71)		0.97	0.39	(0.44–2.14)		1.12	0.3	(0.66–1.9)	
Some college	1.95	0.87	(0.82–4.66)		1.03	0.41	(0.47–2.26)		0.98	0.32	(0.52–1.85)	
Bachelor’s degree	1.88	0.84	(0.79–4.51)		1.04	0.42	(0.47–2.28)		1.11	0.32	(0.63–1.96)	
Post-Bachelor’s degree	1.16	0.53	(0.48–2.82)		0.86	0.38	(0.36–2.02)		1.47	0.6	(0.66–3.28)	
Other	2.23	1.6	(0.55–9.11)		2.35	2.74	(0.24–23.21)			^b^		
**Employment status**				0.686				0.596				0.896
Not employed (includes students and retired)	*ref*				*ref*				*ref*			
Employed	1.11	0.28	(0.67–1.82)		0.9	0.17	(0.62–1.31)		0.98	0.16	(0.71–1.35)	
**COVID-19 risk perception**				<0.001				0.040				0.014
Low COVID-19 risk perception (<25th percentile)	*ref*				*ref*				*ref*			
Medium COVID-19 risk perception (≥25th percentile; <75th percentile)	0.47 *	0.15	(0.24–0.89)		0.74	0.16	(0.48–1.14)		0.79	0.21	(0.47–1.33)	
High COVID-19 risk perception (≤75th percentile)	0.17 **	0.06	(0.09–0.34)		0.53 *	0.13	(0.32–0.87)		0.53 *	0.15	(0.3–0.91)	
**Perception of government measures to respond to the pandemic**				<0.001				<0.001				<0.001
Government measures just right	*ref*				*ref*				*ref*			
Government measures have not been right	2.22 **	0.51	(1.42–3.47)		3.05 **	0.54	(2.16–4.31)		2.28 **	0.35	(1.68–3.08)	
**Request for government aid**				0.155				0.074				0.019
No requests rejected or applied for	*ref*				*ref*				*ref*			
At least one request was rejected	0.71	0.17	(0.44–1.14)		1.61	0.43	(0.95–2.72)		1.73 *	0.4	(1.1–2.73)	
**Perception of government transparency**				0.243				0.146				0.480
Government has been transparent	*ref*				*ref*				*ref*			
Government has not been transparent	1.34	0.33	(0.82–2.17)		1.3	0.24	(0.91–1.86)		1.13	0.2	(0.8–1.6)	
**Misinformation endorsement**				<0.001				<0.001				<0.001
Low misinformation endorsement (<25th percentile)	*ref*				*ref*				*ref*			
Medium-Low (≥25th percentile; <50th percentile)	2.21 *	0.76	(1.13–4.33)		5.08 **	1.45	(2.91–8.88)		3.16 **	0.73	(2.01–4.98)	
Medium-High (≥50th percentile; <75th percentile)	6.23 **	2.1	(3.22–12.07)		14.98 **	4.25	(8.59–26.12)		6.02 **	1.44	(3.77–9.63)	
High misinformation endorsement (≥75th percentile)	7.42 **	2.49	(3.85–14.32)		22.01 **	6.53	(12.31–39.38)		10.20 **	2.64	(6.14–16.95)	

* The marginal test associated with the coefficient produced a *p*-value between 0.01 and 0.05. ** The marginal test associated with the coefficient produced a *p*-value smaller than 0.01. ^a^ Race was not assessed in the Italian survey. ^b^ Two respondents in the Italian survey indicated they had received an “other” level of education–these respondents were excluded from the analysis due to a low cell count.

## Data Availability

The data presented in this study are available on request from the corresponding author.

## Supplementary Materials

The following supporting information can be downloaded at: https://www.mdpi.com/article/10.3390/vaccines10050671/s1. Supplementary Material File S1—Survey Instrument. Supplementary Material Table S1—Detailed Factor Analysis Results by Country.
