# Supplementary material for: Exploring the Association between Misinformation Endorsement, Opinions on the Government Response, Risk Perception, and COVID-19 Vaccine Hesitancy in the US, Canada, and Italy
Source: Vaccines (Basel). 2022 Apr 23;10(5):671. doi: 10.3390/vaccines10050671 (PMC9147457; doi:10.3390/vaccines10050671)
Supplement: Supplementary file 1 [file vaccines-10-00671-s001.zip › Supplementary Material File S1_Survey Instrument.pdf]

Supplementary Material File S1 – Survey Instrument

If you intend to use this questionnaire for your project, please cite the publication and inform the authors by sending an e-mail to preparedness@hsph.harvard.edu

1. **Have you taken the COVID-19 vaccine? (Screening question)**
   - o Yes, I received two doses
   - o Yes, I received one dose and the vaccine I took does not require a second dose
   - o I have received one dose of a two-dose COVID-19 vaccine and I plan to get my second dose
   - o I have received one dose of a two-dose COVID-19 vaccine, but I am not sure if I will get my second dose
   - o I have received one dose of a two-dose COVID-19 vaccine, but I do not plan to get the second dose
   - o Not yet, but I have an appointment scheduled
   - o No, and I do not have an appointment scheduled

2. **What is your sex?**
   - o Male
   - o Female

3. **What is your age?**
   - o 18-24
   - o 25-34
   - o 35-44
   - o 45-54
   - o Over 54

4. **What race/ethnicity do you consider yourself?**
   - o White, Non-Hispanic
   - o Black, Non-Hispanic
   - o Asian, Non-Hispanic
   - o Two or more races
   - o Hispanic
   - o Prefer not to say
   - o Other, please specify: ___________

Supplementary Material File S1 – Survey Instrument

5. **What is the highest level of schooling you have completed?**
   - o Less than high school
   - o High school or equivalent
   - o Some college
   - o Bachelor's degree
   - o Post-graduate degree (i.e. Master, PhD, MD, etc)
   - o Other, please specify: ___________

6. **Select the employment status that best describes your current situation [select one option only]:**
   - o I am working--paid employee
   - o I am working--self-employed
   - o I am not working--on unemployment
   - o I am not working--on paid leave or furloughed
   - o I am not working--searching for work
   - o I am retired
   - o I am not working--on disability or worker's comp
   - o I am not working--and not looking for a job
   - o Other, please specify: ___________

7. **Have you received or requested any of the following forms of support since the start of the COVID-19 pandemic?**

	Not requested or received	Have requested but have not yet received	Have requested but the request was rejected	Received
Unemployment benefits				
Wage support (supplement or replacement while still in employment or short-time working schemes)				
Paid sick leave or paid care leave (for example, for those who had to self-isolate or take care of children or dependent adults)				
State aid to businesses				
Other support from public services to help with living expenses or household needs (e.g. benefits, allowances, vouchers, food)				

Supplementary Material File S1 – Survey Instrument

8. How concerned are you about any of the following situations?

	Very concerned	Somewhat concerned	Not concerned
Contracting COVID-19 at work?<br>(For example: your work settings that is not your home)			
Contracting COVID-19 outside of work?<br>(For example: at the grocery store, when you are using transportation, or in other aspects of your daily life)			
Infecting your family or friends with COVID-19?			

9. Do you feel you are receiving fully transparent information about the COVID-19 situation from your national government officials?

- Not at all
- Moderately transparent information
- Very transparent information
- I do not know

10. I think that most of the measures taken so far by the US government to respond to the COVID-19 pandemic have been:

- Just right
- Excessive
- Not useful
- Counter-productive
- I do not know

11. If you were offered a COVID-19 vaccine - at no cost to you - how likely are you to take it?

- Very likely
- Somewhat likely
- I am not sure
- Somewhat unlikely
- Very unlikely
- I would not take it at the moment but would consider it later on

Supplementary Material File S1 – Survey Instrument

12. How much do you agree or disagree with the following statements?

	Strongly disagree	Disagree	Somewhat disagree	Unsure	Somewhat agree	Agree	Strongly Agree
You cannot get COVID-19 from the vaccine itself							
There are no toxic ingredients in the vaccine that can harm your health							
The vaccine cannot mess up your DNA							
The vaccine cannot cause infertility							
The vaccine cannot cause other diseases							
The fast production of the vaccine did not compromise its safety							
There is no microchip with tracking capabilities inserted in the vaccine							
