# Supplementary material for: Exploring the Association between Misinformation Endorsement, Opinions on the Government Response, Risk Perception, and COVID-19 Vaccine Hesitancy in the US, Canada, and Italy
Source: Vaccines (Basel). 2022 Apr 23;10(5):671. doi: 10.3390/vaccines10050671 (PMC9147457; doi:10.3390/vaccines10050671)
Supplement: Supplementary file 1 [file vaccines-10-00671-s001.zip › Supplementary Material Table S1_Misinformation Endorsement Factor Analysis.pdf.pdf]

Table S1 – Detailed Factor Analysis Results by Country

	Overall	US	Canada	Italy
KMO	0.89	0.92	0.91	0.83
Eigenvalue of retained factor *	3.6	4.13	3.92	2.74
Cumulative Variance	0.51	0.59	0.56	0.39
Cronbach's Alpha	0.84	0.88	0.87	0.73
	Factor Loadings			
**Item**	**Overall**	**US**	**Canada**	**Italy**
You cannot get COVID-19 from the vaccine itself	0.68	0.75	0.73	0.58
There are no toxic ingredients in the vaccine that can harm your health	0.77	0.80	0.77	0.73
The vaccine cannot mess up your DNA	0.76	0.79	0.79	0.69
The vaccine cannot cause infertility	0.75	0.81	0.80	0.61
The vaccine cannot cause other diseases	0.73	0.79	0.76	0.64
The fast production of the vaccine did not compromise its safety	0.68	0.75	0.75	0.51
There is no microchip with tracking capabilities inserted in the vaccine	0.63	0.68	0.61	0.60

* All items across each country loaded onto one factor with an eigenvalue greater than 1
